# Notes and News

**Published:** 1895-02-23

**Authors:** 


### Notes and News.

Collected and Communicated.

Small-pox has again shown itself in Marylebone.

At Tonquin, the Hanoi Hospital has been officially inaugurated. It contains 300 beds, and is stated to be excellent in all sanitary arrangements.

The Duke and Duchess of York have promised to open the new wing of the Sheffield Hospital, and to lay the foundation-stone of other buildings connected with the hospital.

The recently decorated members of the medical profession, Sir John Erichsen, Bart., Sir J. Russell Reynolds, Bart., and Sir John Williams, Bart., are to be entertained by their colleagues and pupils at a banquet at the Criterion Restaurant on March 13th. The chair will be taken by Lord Reay, Vice-President of University College.

The new President of the French Republic has evinced an interest in hospitals early in his official life. He has already visited the St. Antoine Hospital in Paris, to the funds of which he has given £20, to be expended, we learn, on better food for the patients. Such a donation carries with it a certain amount of criticism, which may be more salutary even than the gift itself.

A most interesting ceremony will take place at the annual meeting of the governors of the Queen's Hospital, Birmingham, on Wednesday, the 27th inst. The president, the Right Honourable the Earl of Dudley, will present to Mrs. Johnson a replica of the portrait of Alderman Johnson, which has been painted by Mr. Stanhope Forbes, A.R.A. Mr. Alderman Johnson has devoted many years of his life to the best interests of the Queen's Hospital, Birmingham. He is one of the ablest and most popular of lawyers, and his mayoralty at Birmingham will long be remembered as one of the most successful on record. Mr. Alderman Johnson is a hospital worthy of the first rank, and it is but fitting that his portrait should be hung in the board-room of the Queen's Hospital, as a commemoration of his splendid services to the institution.

The King's College Hospital past and present students' dinner will be held at the Holborn Restaurant on Friday, March 1st, Dr. John Curnow in the chair.

We are told that the Berlin police have adopted a very neat device to effect the difficult task of protecting the public from the sale of quack remedies. When one of these preparations contains a poisonous ingredient they insert an analysis in the paper, and give the actual value, together with the selling price, a comparison which shows such disproportion as to teach the public a lesson.

A gift of $350,000 has been made to the New York College of Physicians and Surgeons by members of the Vanderbilt family to add to the buildings of the Vanderbilt Clinic in connection with the College. The proposed addition will render the Clinic complete in all departments. The Medical School has received a donation of $200,000 from Mr. and Mrs. W. D. Sloane to enlarge the Sloane Maternity Hospital. Mrs. Sloane will equip the hospital completely, and has offered not only to maintain it during her lifetime, but to provide for it on her death.

Over 300 cases of scarlet fever are now under treatment in Hornsey, and the portion of Islington which adjoins it, and, with the exception of a few, they are all traceable to the milk. Inquiries made in Derbyshire, where the milk came from, show that the Notification of Infectious Diseases Act is not in force there. Four families in one village have had the disease, and the parents of children affected have continued their work on the farms. Drs. Clothier and Harris, the medical officers of the districts affected, accompanied by a veterinary expert, have been to Derbyshire, and are now engaged upon a report on the whole matter.

We regret to announce the death of Mr. John Whittaker Hulke, F.R.S., President of the Royal College of Surgeons of England, which took place at his residence in Old Burlington Street, on February 20th, from broncho-pneumonia, which is ascribed to a chill caught in going to the Middlesex Hospital in the middle of one of the coldest nights of the winter. Mr. Hulke, who was born in 1830, was educated at King's College School, and afterwards in the medical department of King's College. In 1855 he was appointed surgeon to the hospital established at Smyrna during the Russian war, and afterwards was attached to the hospital before Sebastopol. He become a Fellow of the College of Surgeons in 1857, and the same year was appointed Assistant Surgeon to King's College Hospital; the next year he was made Assistant Surgeon to Moorfields Eye Hospital; in 1859 he received the Johnsonian Prize for his essay on "Diseases of the Retina," and soon afterwards brought out a treatise on the ophthalmoscope. Although looked upon by many as an ophthalmic surgeon, he contributed largely to general surgical work, and joined Mr. Holmes in editing the third edition of his "System of Surgery." He was made a Fellow of the Royal Society in 1866. He many years ago migrated to Middlesex Hospital, to which at the time of his death he was senior surgeon. He was a well-known and frequent attendant at the various medical societies, of several of which he had been president. Mr. Hulke, however, eminent as he was in surgery, was not a surgeon only. In comparative anatomy, in botany, and in geology, he had made his mark, and was at one time President of the Geological Society; he was an artist in water-colours, in clay, and in marble; and was an enthusiastic fisherman, spending most of his holidays by a Scottish trout stream.

